# Smartphone addiction and its impact on quality of sleep and academic performance among nursing students. Institutional based cross-sectional study in Western Rajasthan (India)* ** ***

**DOI:** 10.17533/udea.iee.v41n2e11.

**Published:** 2023-08-24

**Authors:** Nipin Kalal, N Sabari Vel, Sonam Angmo, Sukhi Choyal, Sunaina Bishnoi, Subeeta Dhaka, Suman Rulaniya, Surbhi Banswal

**Affiliations:** 1 Assistant Professor. College of Nursing, All India Institute of Medical Sciences, Jodhpur, Raj, India. Email: kalalnipin@gmail.com. https://orcid.org/0000-0002-1392-1787 All India Institute Of Medical Sciences College of Nursing All India Institute of Medical Sciences Jodhpur Raj India kalalnipin@gmail.com; 2 Nursing Tutor. College of Nursing, All India Institute of Medical Sciences, Jodhpur, Raj, India. Email: id-sabari123vel@gmail.com https://orcid.org/0000-0002-3187-2683 All India Institute Of Medical Sciences College of Nursing All India Institute of Medical Sciences Jodhpur Raj India id-sabari123vel@gmail.com; 3 B.Sc. Nursing. College of Nursing, All India Institute of Medical Sciences, Jodhpur, Raj, India. Email: angmoyourock123@gmail.com https://orcid.org/0000-0002-0927-0950 All India Institute Of Medical Sciences College of Nursing All India Institute of Medical Sciences Jodhpur Raj India angmoyourock123@gmail.com; 4 B.Sc. Nursing. College of Nursing, All India Institute of Medical Sciences, Jodhpur, Raj, India. Email: sukhichoyal180@gmail.com https://orcid.org/0000-0001-5257-9008 All India Institute Of Medical Sciences College of Nursing All India Institute of Medical Sciences Jodhpur Raj India sukhichoyal180@gmail.com; 5 B.Sc. Nursing. College of Nursing, All India Institute of Medical Sciences, Jodhpur, Raj, India. Email: bishnoisunaina29@gmail.com https://orcid.org/0000-0002-4406-5987 All India Institute Of Medical Sciences College of Nursing All India Institute of Medical Sciences Jodhpur Raj India bishnoisunaina29@gmail.com; 6 B.Sc. Nursing. College of Nursing, All India Institute of Medical Sciences, Jodhpur, Raj, India. Email: dhakasubhi22@gmail.com https://orcid.org/0000-0003-2547-9321 All India Institute Of Medical Sciences College of Nursing All India Institute of Medical Sciences Jodhpur Raj India dhakasubhi22@gmail.com; 7 B.Sc. Nursing. College of Nursing, All India Institute of Medical Sciences, Jodhpur, Raj, India. Email: sumanrulaniya5@gmail.com https://orcid.org/0000-0002-6578-661X All India Institute Of Medical Sciences College of Nursing All India Institute of Medical Sciences Jodhpur Raj India sumanrulaniya5@gmail.com; 8 B.Sc. Nursing. College of Nursing, All India Institute of Medical Sciences, Jodhpur, Raj, India. Email: surbhibanswal99@gmail.com https://orcid.org/0000-0002-0927-0950 All India Institute Of Medical Sciences College of Nursing All India Institute of Medical Sciences Jodhpur Raj India surbhibanswal99@gmail.com

**Keywords:** academic performance, sleep quality, smartphone, students, nursing, cross-sectional studies, rendimiento académico, calidad del sueño, teléfono inteligente, estudiantes de enfermería, estudios transversales, desempenho académico, qualidade do sono, smartphone, estudantes de enfermagem, estudos transversais

## Abstract

**Objective:**

To explore the smartphone addiction and its impact on quality of sleep and academic performance among the nursing students.

**Methods:**

A descriptive cross-sectional study was conducted among the nursing students (*n=*160) in tertiary care teaching hospital in western Rajasthan (India) by using standardized Smartphone Addiction Scale Short Version (SAS-SV), the quality of sleep was assessed by standardized Pittsburg’s Sleep Quality Index scale (PSQI) and academic performance was assessed by self -made Academic Performance Scale.

**Results:**

In this study 38.1 % students were having moderate smartphone addiction. The smartphone addiction is directly associated with hours daily spend on smartphone (*p*<0.001), time check smartphone after wake up in the morning (*p*<0.001), and frequency of smartphone pickups in a day (*p*=0.003) of students. The quality of sleep is inversely associated with duration of smartphone use (*p=*0.004), hours daily spend on smartphone (*p*=0.002), time check smartphone after wake up in morning (*p*=0.010), of students The academic performance is significantly associated with hours daily spend on smartphone (*p*=0.003), time check smartphone after wake up in morning (*p*=0.001), and frequency of smartphone pickups in a day (*p*=0.015) of students.

**Conclusion:**

A high proportion of nursing students have moderate smartphone addiction. This addiction was associated with an increased risk of poor sleep quality and poor academic performance. Educational activities on the healthy use of smartphones are needed in the studied group.

## Resumen

**Objetivo:**

Explorar la adicción a los teléfonos inteligentes y su impacto en la calidad del sueño y el rendimiento académico entre los estudiantes de enfermería.

**Métodos:**

Se realizó un estudio descriptivo transversal entre los estudiantes de enfermería (*n*=160) en el hospital universitario de atención terciaria en el oeste de Rajastán (India). Se empleó la escala estandarizada Smartphone Addiction Scale Short Version (SAS-SV), la calidad del sueño se evaluó mediante el Pittsburg's Sleep Quality Index (PSQI) y el rendimiento académico se evaluó mediante la Escala de Rendimiento Académico de elaboración propia.

**Resultados:**

En este estudio, el 38.1% de los estudiantes presentaban adicción moderada a los teléfonos inteligentes. Esta adicción se asoció con las horas diarias dedicadas a los teléfonos inteligentes (*p*<0.001), el tiempo de revisión de los teléfonos después de despertarse por la mañana (*p*<0.001), y la frecuencia de uso durante el día (*p*=0.003). La calidad del sueño se asoció inversamente con los años de uso del teléfono inteligente (*p*=0.004), las horas diarias de uso (*p*=0.002) y el tiempo de revisión del teléfono después de despertarse por la mañana (*p*=0.01). El rendimiento académico también se asoció en forma inversa con las horas diarias de uso del teléfono (*p*=0.003), el tiempo de revisión del teléfono después de despertarse por la mañana (*p*=0.001), y la frecuencia de uso del teléfono en un día (*p*=0.015).

**Conclusión:**

Una alta proporción de estudiantes de enfermería tienen adicción moderada a los teléfonos inteligentes. Esta adicción se asoció a un mayor riesgo de mala calidad del sueño y de bajo rendimiento académico. Es necesario llevar a cabo actividades educativas sobre el uso saludable de los teléfonos inteligentes en el grupo estudiado.

## Resumo

**Objetivo:**

Explorar o vício em smartphones e seu impacto na qualidade do sono e no desempenho acadêmico entre estudantes de enfermagem.

**Métodos:**

Um estudo transversal descritivo foi realizado entre estudantes de enfermagem (*n* = 160) em um hospital universitário terciário no Oeste Rajasthan (Índia). Foi utilizada a escala padronizada de dependência de smartphones, Scale Short Version (SAS-SV), a qualidade do sono foi avaliada usando o Pittsburg's Sleep Quality Index (PSQI) e desempenho acadêmico foi avaliado por meio da Escala de Desempenho Acadêmico de nossa própria elaboração.

**Resultados:**

Neste estudo, 38.1% dos alunos apresentaram dependência moderada de smartphones. Esse vício foi associado a horas diárias gastas em smartphones (*p*<0.001), tempo verificando telefones após acordar pela manhã (*p*<0.001) e frequência de uso durante o dia (*p*=0.003). A qualidade do sono foi inversamente associada à duração do uso do smartphone (*p*=0.004), horas diárias de uso (*p*=0.002) e tempo gasto checando o telefone após acordar pela manhã (*p*=0.01). O desempenho acadêmico também foi inversamente associado com horas diárias de uso do telefone (*p*=0.003), tempo gasto verificando o telefone após acordar pela manhã (*p*=0.001) e frequência de uso do telefone num dia (*p*=0.015).

**Conclusão:**

Uma alta proporção de estudantes de enfermagem tem um vício moderado em smartphones. Esse vício foi associado a um risco aumentado de má qualidade do sono e baixo desempenho acadêmico. É necessária a realização de atividades educativas sobre o uso saudável de smartphones no grupo estudado.

## Introduction

In the modern world highly revolving technology, the use of smartphones has become a very necessary gadget. According to studies, there are 5.22 billion smartphone users in the world. This accounts for 66.6 % of the global population data according to 2021. Moreover, the number of smartphone users has increased by 1.8% from January 2020 to January 2021 during the covid-19 pandemic period.^1^ On average, a person spends 6 hours and 54 minutes on the internet. A person accesses their phone 160 times a day, or once every 9 minutes, according to 2020 statistics. In class, 49 percent of pupils are using their smartphones as a distraction. As a result, 76.19 percent of professors think that students' use of smartphones during class is distracting.^1^ It has been seen that students preoccupied while on a smartphone, get decreased awareness of what is happening around their surroundings, he/she is not aware of what she is speaking, doing, or eating as their eyes and mind is focusing on their smartphone. According to Hassan et al. said that utilised for online learning or other types of learning, smartphones have a detrimental impact on students' academic performance. Students spend the bulk of their time on their smartphones, which severely lowers their ability to communicate and work in groups.^2^ People use their smartphones more frequently during COVID -19 periods because all universities provide online courses. The majority of people have demonstrated smartphone addiction. 69.7% of respondents said they could not focus on their homework at home, 97.6% of them said they had nomophobia, and 45.1% said they were addicted to their smartphones.^3^

Overuse of smartphones reduces sleep quality, which has an impact on students' learning, focus, memory, and ability to make decisions.^4^ Students at universities require enough, high-quality sleep for optimal academic success. Despite having a strong understanding of the value of sleep and how it affects their academic performance, students often neglect their sleep schedules.^5^

Sleep quality has significant impact on cognitive ability and physical strength, the consequences of poor sleep quality and also have some serious problem such as depression, impaired work performance, and poor overall quality of life.^6^ Students spend more time browsing social media and gaming apps on their smartphones, which results in excessive smartphone use, time spent on the device other than for studying, and low academic achievement.^7^ The nursing profession is the backbone of the health care system and nursing students are one of the upcoming healthcare givers whose own health wellbeing is very important to provide health services. This topic is chosen because very few studies are performed in India on undergraduate nursing students. This study is giving a picture depicting addition of smartphone and its impact on quality of sleep and academic performance in student nurses who are undoubtedly a pillar of a successfully working community healthcare system.

## Methods

In this cross-sectional descriptive research design, we evaluated the mobile addiction and its impact on quality of sleep and academic performance among nursing students in from March to May 2022. The study participants were selected by using the total enumerative sampling technique. The sample size calculation is based on a similar study found through the review of literature done by Noruzi Kuhdasht^8^ where d=confidence interval, that is 7% (0.07) t=1.96, it is standard deviation score for 95% set interval *p*=assumed or estimated proportion so p=0.24 q=(1-p) = 1-0.24 q=0.76, the sample size was calculated as 143, while considering 10% non-response rate, a total sample size of 160 participants were considered for present study. 

Nursing students who have smartphone and using for last 6 months were included in the study. Those Students who already suffer from sleep issues and are using sleep medicine and not available at the time of data collection were excluded from the study. Based on the objective of the study self-structured questionnaire and standard tools were used to gather information regarding smartphone addiction, quality of sleep and academic performance. It consists of following part: (i) *Demographic data*: It includes 10 questions related to socio demographic data such as age, gender, education, living area, duration of smartphone use, daily hours spent on smartphone, when you wake up in the morning at what time you check your smartphone, use of smartphone, frequency of smartphone use, and family history of sleep disorder; (ii) *The Smartphone addiction scale short version (SAS-SV)*: this scale is a self-reported, standardised questionnaire that measures smartphone addiction. Short-version of SAS consists of 10 items on six-point Likert’s scale named with strongly agree to strongly disagree. All 10 items are positive items. The Cronbach’s alpha reliability of SAS-SV is 0.911,^9^ *(iii) Pittsburgh Sleep Quality Index (PSQI)*: standardized and self-rated questionnaire which assesses sleep quality and disturbances over month time interval. Quality of sleep includes quantitative aspects of sleep, such as sleep durations, sleep latency or number of arousals, as well as more purely subjected aspects, such as depth or restfulness of sleep. The PSQI contains 19 self-rated questions which are included in the scoring the 19 self-rated items are combined to form seven component scores. The index has score for 7 items: quality of sleep, delay of falling asleep, effective duration of sleep, sleep efficacy, sleep disorders, needed number of sleep-inducing pills, and day-time performance. Each item has a score of 0 to 3 (0 indicates no difficulty and 3 indicates severe difficulty) and these items form overall score of 0 to 21. The reliability of PQSI is 0.726,^10^ *(iv) Self-structured academic performance scale:* the self-structured academic performance scale was prepared by investigators to assess the academic performance among the nursing students. This tool consisted of 9 items of 3 points Likert’s scale Scoring: For positive items: always (3), sometimes (2), never (1). If the student score between 9-15, will be having good academic performance and if score is between 22-27, considered as poor academic performance. The Cronbach’s alpha reliability of the scale is 0.887.

Ethical approval was taken from the institutional ethical committee to conduct the study. The certificate Reference Number is AIIMS/IEC/2022/3890 dated: 25 February 2022. The written permission and informed consent were taken from the principal and students respectively. Confidentiality and anonymity of the students and data collected were maintained throughout the study. To respect the ethical codes, the institution remained anonymous in this study. Data were coded and entered an excel sheet and analysed using SPSS software version 16 (IBM Inc, Armonk, New York, USA). Descriptive statistics, including frequency, percentage, mean, and standard deviation, were used for central and deviation indexes. Inferential statistics were used to find the level of association between the selected personal variables and smartphone addiction, quality of sleep and academics performance. 

## Result

In the present study, we approached 160 nursing students where 44.4% of students were in the age group of 20-21 years. Most of the students 98.1% were females in this study. The professional course of 87.5% of students was B.Sc. (Hons.) nursing and 12.5% of students were M.Sc. 30 nursing. The Majority of students 86.2% were living in a hostel and 13.8% of students were living at home with parents. Half of the students 51.2% were using smartphones for 1 to 3 years of duration. The majority 37.5% of students spent 1 to 3 hours daily on smartphones. Majority of 31.9% students were check smartphone within 5 minutes after wake up in morning. Majority of 43.8% student’s pickups smartphone 11-20 times per day followed by 19.1% were more than 30 times. The majority of 92.5% students were use the smartphone for academic uses followed by 91.8% were social media. Most of the students 92.5% had no family history of sleep disorder. (Table 1)

**Table 1:** Socio-demographic characteristics of 160 nursing students

Demographic variable	Frequency (%)
Age (Years)	
18-19	5 (3.1)
20-21	71 (44.4)
22-23	67 (41.9)
24 and more	17 (10.6)
Gender	
Male	3 (1.9)
Female	157 (98.1)
Professional course	
B.Sc. nursing	140 (87.5)
M.Sc. nursing	20 (12.5)
Place of living	
Living in hostel	138 (86.2)
Living with parents (home)	22 (13.8)
Duration of smartphone use	
Less than 1 year	6 (3.8)
1 to 3 years	82 (51.2)
4 to 6 years	41 (25.6)
More than 6 years	31 (19.4)
Hours daily spend on smartphone	
Less than 1 hour	9 (5.6)
1 to 3 hours	60 (37.5)
4 to 5 hours	55 (34.4)
More than 5 hours	36 (22.5)
Time check smartphone after wake up in morning	
Within 5 minutes	51 (31.9)
Within 6 to 30 minutes	49 (30.6)
Within 31 to 60 minutes	31 (19.4)
More than 60 minutes	29 (18.1)
Frequency of smartphone pickups in a day	
Less than 10 times per day	30 (18.8)
11-20 times per day	70 (43.8)
21-30 times per day	29 (18.1)
More than 30 times per day	31 (19.4)
Use of mobile phone*	
Academic use	148 (92.5)
Social media	147 (91.8)
Playing game	25 (15.6)
Listening to music	126 (78.7)
Speaking to family and friends	141 (88.1)
Family history of sleep disorder	
Yes	12 (7.5)
No	148 (92.5)

Table 2 shows 38.1 % students were having moderate smartphone addiction. This addiction among student’s ranges from 10-45 with a mean of 26.44±8.67. Also 42.5% of students had poor quality of sleep. Quality of sleep among students range from 0 to 13 with a mean 4.73±3.10. Table 2 also shows that 5% of students were having poor academic performance; this scale in students’ ranges from 9 to 24 with a mean of 14.78±3.60.

**Table 2:** Levels of smartphone addiction, quality of sleep and academic performance among 160 nursing students

Scale	Score	Frequency (%)	Mean± SD
Smartphone addiction			
Mild	<30	99 (61.9)	20.91±5.88
Moderate	31-45	61 (38.1)	35.39±3.33
Total scale			26.44±8.67
Quality of sleep			
Good	0-5	92 (57.5)	2.46±1.44
Poor	6-21	68 (42.5)	7.79±1.87
Total			4.73±3.10
Level of academic performance			
Good	9-15	93 (58.1)	12.23±1.89
Average	16-21	59 (36.9)	17.76±1.57
Poor	22-27	8 (5.0)	22.37±0.74
Total			14.78±3.60

A significant positive correlation was seen between smartphone addiction and quality of sleep (r=0.45; *p*<0.001) which states that those students who had greater smartphone addiction had poor quality of sleep and also showed a significant positive correlation between smartphone addiction and academic performance (r=0.5; *p*<0.001), which states that those students who had smartphone addiction they had poor academic performance. A significant positive correlation was seen between quality of sleep and academic performance (r=0.32; *p*<0.001) states that those students who had poor quality of sleep were having poor academic performance.

It was found that smartphone addiction and academic performance significant association between hours daily spend on smartphone, time check smartphone after wake up in morning and frequency of smartphone pickups in a day. Furthermore, quality of sleep is significantly associated with duration of smartphone use, hours daily spend on smartphone, time check smartphone after wake up in morning and family history of sleep disorder. (Table 3)

**Table 3:** Association of smartphone addiction, quality of sleep, and academic performance with demographic variables (*n*=160)

Demographic Variables	Smartphone addiction		***p*-value**	Quality of sleep		***p*-value**	Academic performance			***p*-value**
	Mild f (%)	Moderate/ Severe f (%)		Good f (%)	Poor f (%)		Good f (%)	Average f (%)	Poor f (%)	
Age (Years)			0.13			0.06				0.42
18-19	5 (3.1)	0 (0)		5 (3.1)	0 (0)		5 (3.1)	0	0	
20-21	47 (29.4)	24 (15)		42 (26.2)	29 (18.1)		41 (25.6)	27 (16.9)	3 (1.9)	
22-23	39 (24.4)	28 (17.5)		39 (24.4)	28 (17.5)		35 (21.9)	28 (17.5)	4 (2.5)	
Above 23	8 (5)	9 (5.6)		6 (3.8)	11 (6.9)		12 (7.5)	4 (2.5)	1 (0.6)	
Gender			0.30			0.13				0.33
Male	1 (0.6)	2 (1.2)		3 (1.9)	0 (0)		3 (1.9)	0	0	
Female	98 (61.2)	59 (36.9)		89 (55.6)	42.5)		90 (56.2)	59 (36.9)	8 (5)	
Professional course			0.85			0.80				0.49
B.Sc. nursing	87 (54.4)	53 (33.1)		81 (50.6)	59 (36.9)		79 (49.4)	54 (33.8)	7 (4.4)	
M.Sc. nursing	12 (7.5)	8 (5)		11 (6.9)	9 (5.6)		14 (8.8)	5 (3.1)	1 (0.6)	
Place of living			0.85			0.27				0.2
Living in hostel	85 (53.1)	53 (33.1)		77 (48.1)	61 (38.1)		82 (51.2)	48 (30)	8 (5)	
Living with parents	14 (8.8)	8 (5)		15 (9.4)	7 (4.4)		11 (6.9)	11 (6.9)	0 (0)	
Duration of smartphone use			0.14			0.001				0.249
< 1 year	5 (3.1)	1 (0.6)		6 (3.8)	0 (0)		6 (3.8)	0 (0)	0 (0)	
1 to 3 years	53 (33.1)	29 (18.1)		56 (35)	26 (16.2		49 (30.6)	29 (18.1)	4 (2.5)	
4 to 6 years	27 (16.9)	14 (8.8)		18 (11.2)	23 (14.4		24 (15)	14 (8.8)	3 (1.9)	
More than 6 years	14 (8.8)	17 (10.6)		12 (7.5)	19 (11.9)		14 (8.8)	16 (10)	1 (0.6)	
Hours daily spend on smartphone			<0.001			0.003				0.003
<1 hour	6 (3.8)	3 (1.9)		6 (3.8)	3 (1.9)		8 (5)	0 (0)	1 (0.6)	
1 to 3 hours	51 (31.9)	9 (5.6)		43 (26.9)	17 (10.6)		44 (27.5)	14 (8.8)	2 (1.2)	
4 to 5 hours	30 (18.8)	25 (15.6)		31 (19.4)	24 (15)		28 (17.5)	24 (15)	3 (1.9)	
6 and more hours	12 (7.5)	24 (15)		12 (7.5)	24 (15)		13 (8.1)	21 (13.1)	2 (1.2)	
Time check smartphone after wake up in morning			<0.001*			0.002*				0.001
0 to 5 minutes	24 (15)	27 (16.9)		22 (13.8)	29 (18.1		23 (14.4)	24 (15)	4 (2.5)	
6 to 30 minutes	25 (15.6)	24 (15)		24 (15)	25 (15.6		21 (13.1)	25 (15.6)	3 (1.9)	
31 to 60 minutes	26 (16.2)	593.1)		24 (15)	7 (4.4)		25915.6)	6 (3.8)	0 (0)	
61 and more minutes	24 (15)	5 (3.1)		22 (13.8)	7 (4.4)		24 (15)	4 (2.5)	1 (0.6)	
Frequency of smartphone pickups in a day			0.003*			0.42				0.015
<10 times per day	21 (13.1)	9 (5.6)		15 (9.4)	15 (9.4)		23 (14.4)	6 (3.8)	1 (0.6)	
11-20 times per day	51 (31.9)	19 (11.9)		44 (27.5)	26 (16.2)		44 (27.5)	24 (15)	2 (1.2)	
21-30 times per day	16 (10)	13 (8.1)		18 (11.2)	11 (6.9)		16 (10)	12 (7.5)	1 (0.6)	
31 and more times per day	11 (6.9)	20 (12.5)		15 (9.4)	16 (10)		10 (6.2)	17 (10.6)	4 (2.5)	
Family history of sleep disorder			0.37			0.018*				0.69
Yes	6 (3.8)	6 (3.8)		3 (1.9)	9 (5.6)		7 (4.4)	5 (3.1)	0 (0)	
No	93 (58.1)	55 (34.4)		89 (55.6)	59 (36.9)		86 (53.8)	54 (33.8)	8 (5)	

## Discussion

Nowadays, a very large number of populations especially the young people is excessively spending their time and enjoying smartphone. However, in recent literature has been revealed that the excessive use of smartphone can influence daily life activities and may cause addiction.^11^ This study was conducted among 160 nursing students to explore smartphone addiction and its impact on quality of sleep and academic performance among nursing students.

### Students and use of smartphone

The current study result shows that, 37.5% students spent 1-3 hours daily on mobile. These findings are in resemblance with another similar study.^12^ This study showed that the 91.8% students use mobile for social media These results are in line with other studies from Turkey,^13^ Germany^14^ claiming that most students are heavily using the platform of social media on mobile. 

Another study involving college students in Hainan showed a 40.5% mobile phone addiction rate.^15^ Consequently, the finding is complementary to our study finding where 38.1% students are moderately addicted with smartphone.

### Smartphone addiction and quality of sleep

Current study shows 61.9% of students were having mild smartphone addiction and 38.1 % of students were having moderate smartphone addiction. It supported by another study which out of 100 students, 54% of students were found to be not addicted, while 46% of were addicted to smartphone.^16^ The present study shows smart phone addiction and quality of sleep it shows 42.5% of students had poor quality of sleep. These findings are similar to the findings of a previous study, which out of 224 students, 63.3% of students were found poor quality of sleep. Poor sleep quality may experience more stress and lack of physical and mental health. The smartphone addiction significantly correlated with poor quality of sleep and this finding is complementary to other study which a positive correlation between smartphone addiction score (SV) and PSQI score.^17^

Lemolas *et al.* have evaluated the relationship between smartphones, and sleep disturbances in adolescents and found association between smartphone and impaired sleep.^18^ This finding are resemble with our study. Sei *et al.* reported that large proportion of participants disclosed poor sleep (61.6%). This finding is congruent to our study where 42.5% students had poor quality sleep.^19^ The current study result shows that, quality of sleep is significantly associated with duration of smartphone use, hours daily spend on smartphone, time check smartphone after wake up in morning and family history of sleep disorder. These findings are in resemblance with another similar study conducted by Mortazavi et al. determined a statistically significant relationship between the number of sleeping problems and the amount of time they used mobile phones.^20^

### Smartphone addiction and academic performance

The present study shows the mean of smartphone addiction and academic performance are 26.44±8.677 and 14.78±3.604. The smartphone addiction significantly correlation with academic performance. In other similar study mean impact on academic and smartphone dependency score are 19.92±7.01 and 48.58±11.46.^21^ The nursing students are faced academic pressure due to overload of assignment and stressful environment, so they are prone to develop social anxiety and mobile phone addiction it leads to lack of academic performance. The academic performance is significantly associated with hours daily spend on smartphone (*p*=0.003), time check smartphone after wakeup in morning (*p*=0.001), and frequency of smartphone pickups in a day (*p*=0.015) of students. This finding is congruent by another study conducted in Korea which time spent daily on weekends, frequency of use on weekdays, purpose of use, Nomophobia (NMP), and smartphone addiction (SA) were significantly associated with low perceived academic performance (PAP).^22^ Further, incorrect and excessive mobile phone use may lead to an academic burnout,^23^ this finding are aligns with our study where students had poor academic performance who are addicted with smartphone.

The study of Chaudhury and Tripathy that examined the relationship between smartphone addiction and academic performance^24^ concluded that high addiction to smartphones lowers academic performance. This finding had an agreement with our study. Our study demonstrated a significant positive correlation between quality of sleep and academic performance (*p*<0.001). Other two studies revealed similar finding that there is a significant association between sleep quality and academic performance.^25^^,^^26^

## Conclusion

Dependency on gadgets among students has been increasing nowadays, as they spend crucial hours of their day-to-day life on such appliances, which in the long run causes problems in academics and sleep pattern and social life. The present study concluded that increased frequency of smartphone usage among college students leaves a serious impact on their academics and quality of sleep. Therefore, institutions need to develop guidelines and protocols regarding smartphone usage and should also conduct educational activities on the healthy use of smartphones.
